# (3*R*,4*S*,5*S*)-4-Hydr­oxy-3-methyl-5-[(2*S*,3*R*)-3-methyl­pent-4-en-2-yl]tetra­hydro­furan-2-one

**DOI:** 10.1107/S1600536808021181

**Published:** 2008-07-16

**Authors:** Annika Becker, Markus Schürmann, Hans Preut, Martin Hiersemann

**Affiliations:** a Fakultät Chemie, Technische Universität Dortmund, Otto-Hahn-Strasse 6, 44221 Dortmund, Germany

## Abstract

The title compound, C_11_H_18_O_3_, was synthesized to prove the relative configuration of the corresponding acyclic C1—C8 stereopentade. It crystallizes with two mol­ecules in the asymmetric unit, which show only slight differences. The mol­ecules are linked *via* O—H⋯O hydrogen bonds, resulting in two crystallographically independent chains of mol­ecules propagating in the *a*-axis direction. The absolute configuration was known from the synthesis.

## Related literature

For related literature, see: Abraham, Körner & Hiersemann (2004 ▶); Abraham, Körner, Schwab & Hiersemann (2004 ▶); Corey & Snider (1972 ▶); Evans *et al.* (1981 ▶, 1999 ▶); Körner & Hiersemann (2006 ▶, 2007 ▶); Pollex & Hiersemann (2005 ▶).
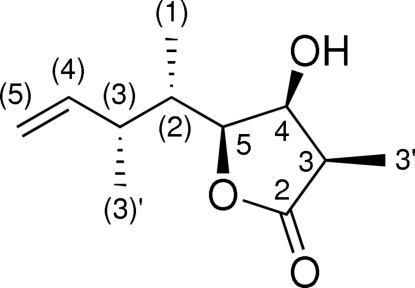


## Experimental

### 

#### Crystal data

C_11_H_18_O_3_
*M* *_r_* = 198.25
Monoclinic, 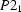

*a* = 6.2934 (13) Å
*b* = 16.411 (3) Å
*c* = 11.607 (2) Å
β = 95.46 (3)°
*V* = 1193.4 (4) Å^3^
*Z* = 4
Mo *K*α radiation
μ = 0.08 mm^−1^
*T* = 291 (1) K
0.30 × 0.28 × 0.20 mm

#### Data collection

Nonius KappaCCD diffractometer
Absorption correction: none
9263 measured reflections
2268 independent reflections
1228 reflections with *I* > 2σ(*I*)
*R*_int_ = 0.026

#### Refinement

*R*[*F* ^2^ > 2σ(*F* ^2^)] = 0.036
*wR*(*F* ^2^) = 0.080
*S* = 1.06
2268 reflections
262 parameters
1 restraint
H-atom parameters constrained
Δρ_max_ = 0.14 e Å^−3^
Δρ_min_ = −0.10 e Å^−3^

### 

Data collection: *COLLECT* (Nonius, 1998 ▶); cell refinement: *DENZO* and *SCALEPACK* (Otwinowski & Minor, 1997 ▶); data reduction: *DENZO* and *SCALEPACK*; program(s) used to solve structure: *SHELXS97* (Sheldrick, 2008 ▶); program(s) used to refine structure: *SHELXL97* (Sheldrick, 2008 ▶); molecular graphics: *SHELXTL-Plus* (Sheldrick, 2008 ▶); software used to prepare material for publication: *SHELXL97* and *PLATON* (Spek, 2003 ▶).

## Supplementary Material

Crystal structure: contains datablocks I, global. DOI: 10.1107/S1600536808021181/hb2741sup1.cif

Structure factors: contains datablocks I. DOI: 10.1107/S1600536808021181/hb2741Isup2.hkl

Additional supplementary materials:  crystallographic information; 3D view; checkCIF report

## Figures and Tables

**Table 1:** Hydrogen-bond geometry (Å, °)

*D*—H⋯*A*	*D*—H	H⋯*A*	*D*⋯*A*	*D*—H⋯*A*
O2—H2⋯O3^i^	0.82	2.03	2.821 (3)	163
O2′—H2′⋯O3′^i^	0.82	1.96	2.771 (3)	171

Symmetry code: (i) 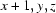.

**Fig. 1.**
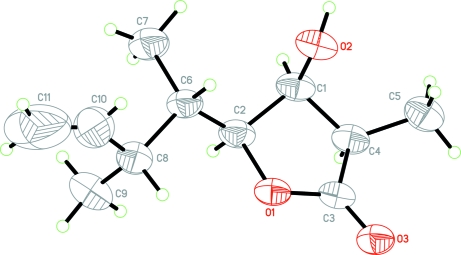
: The molecular structure of molecule one of (I), with displacement ellipsoids for the non-hydrogen atoms shown at the 30% probability level.

**Fig. 2.**
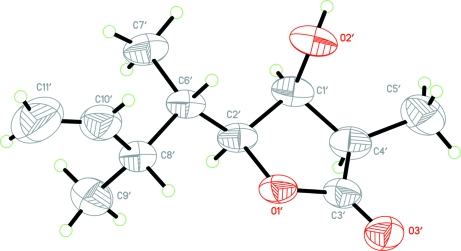
: The molecular structure of molecule two of (I), with displacement ellipsoids for the non-hydrogen atoms shown at the 30% probability level.

## supplementary crystallographic information

### Comment

The title compound, (I), was synthesized using a catalytic asymmetric Claisen rearrangement (Abraham *et al.*, 2004*a*; Abraham *et al.* 2004*b*; Pollex & Hiersemann, 2005; Körner & Hiersemann, 2006; Körner & Hiersemann, 2007), a diastereoselective reduction with K-Selectride (Körner & Hiersemann, 2006; Körner & Hiersemann, 2007), and an aldol addition under modified Evans conditions (Evans *et al.*, 1981). In order to verify the relative configuration of the major diastereomer of the obtained aldol adduct (dr = 7/3), 4-(*tert*-butyldimethylsilyloxy)-3-hydroxy-2,5,6-trimethyloct-7-enoyl)-4-isopropyloxazolidin-2-one, (II), a γ-lactone, (I), was prepared by removal of the silyl protecting group (Corey *et al.*, 1972) and subsequent *in situ* lactonization. Fig. 1 depicts the structure of the isolated major diastereomer (I). The configuration of the chiral C atoms in (I) can be attributed to the stereochemical course of the aldol addition (C3 *R* and C4 *S*), the diastereoselective reduction with K-Selectride (C5 *S*), and the catalytic asymmetric Claisen rearrangement (C2 *S* and C3 *R*) using the chiral Lewis acid [Cu{(*S*,*S*)-*tert*-Butyl-box}](H_2_O)_2_(SbF_6_)_2_ (Evans *et al.*, 1999).

There are two molecules of (I) in the asymmetric unit (Figs. 1 and 2) with similar conformations. In the crystal, the molecules interact via O—H···O hydrogen bonds (Table 1) to form two independent chains, both propagating in [100].

### Experimental

The title compound, (I), was synthesized from the corresponding *anti*-aldol adduct, (II), using tetrabutylammonium fluoride (TBAF) (Corey *et al.*, 1972) for the removal of the silyl protecting group. The subsequent lactonization proceeded *in situ*.

TBAF (1 *M* in tetrahydrofuran, 0.82 ml, 3.0 eq) was added to a solution of the diastereomeric mixture of (II) (dr = 7/3, 120 mg, 0.27 mmol, 1.0 eq) in dry tetrahydrofuran (2 ml) at 273 K. The mixture was stirred at 273 K for 15 min and then at 298 K for 30 min. The reaction was quenched by the addition of saturated aqueous NaHCO_3_ solution. The phases were separated, and the aqueous phase was extracted with CH_2_Cl_2_. The combined organic layers were dried over MgSO_4_ and concentrated under reduced pressure. Flash chromatography (isohexane/ethyl acetate 20/1 to 10/1) afforded (I) as a single diastereomer and additionally a mixture of (I) and the minor diastereomer with an overall yield of 72% (38.7 mg, 0.195 mmol) as colourless crystals. Single crystals of (I) were obtained by vapor diffusion recrystallization technique from isohexane and ethyl acetate to yield colourless cuboids: mp 412 K; R*_f_* 0.33 (cyclohexane/ethyl acetate 2/1); ^1^H NMR (CDCl_3_, 400 MHz, δ): 0.84 (*d*, *J* = 7.0 Hz, 3H, (1)-H), 0.97 (*d*, *J* = 7.0 Hz, 3H, (3)'-H), 1.27 (*d*, *J* = 7.3 Hz, 3H, 3'-H), 1.85 (*d*, *J* = 4.8 Hz, 1H, –OH) overlapped by 1.89 (*br*. *s*), 2.19 (*dqd*, *J* = 10.6, 7.0, 3.0 Hz, 1H, (2)-H), 2.73 (*dq*, *J* = 4.8, 7.3 Hz, 1H, 3-H) overlapped by 2.62 - 2.72 (*m*, 1H, (3)-H), 4.07 (*dd*, *J* = 10.6, 3.0 Hz, 1H, 5-H), 4.37 (*dd*, *J* = 7.3, 4.8 Hz, 1H, 4-H), 5.03 (dd, ^3^J(E) = 17.0 Hz, ^2^J = 1.3 Hz, 1H, (5)-H), 5.04 (*dd*, ^3^*J*(*Z*) = 11.0 Hz, ^2^*J* = 1.3 Hz, 1H, (5)-H), 5.84 (*ddd*, ^3^*J*(*E*) = 17.0 Hz, ^3^*J*(*Z*) = 11.0 Hz, ^3^*J* = 6.3 Hz, 1H, (4)-H); ^13^C NMR (CDCl_3_, 100 MHz, δ): 8.4 (CH_3_), 9.7 (CH_3_), 12.3 (CH_3_), 35.7 (CH), 36.9 (CH), 42.7 (CH), 71.7 (CH), 84.3 (CH), 114.3 (CH_2_), 142.7 (CH), 178.4 (C); IR (cm^-1^): 3435(*br*, *nm*) (ν O—H, OH in H-bridges), 3085(*w*) 3020(*w*) (ν C—H, olefin), 2970(*m*) 2925(*m*) 2855(*s*) (ν_as,s_ C—H, CH_2_, CH_3_, CH), 1740(*s*) (ν C=O, lactone), 1640(*w*) (ν C=C), 1465(*m*) (δ_as_ C—H, CH_3_, CH_2_), 1380(*m*) (δ_s_ C—H, CH_3_); Anal. Calcd. for C_11_H_18_O_3_: C, 66.6; H, 9.2; Found: C, 66.5; H, 9.3; [α]_D_^20^ +2.2 (c 0.472, CHCl_3_).

### Refinement

Anomalous dispersion was negligible and Friedel pairs were merged before refinement. The H atoms were geometrically placed (C—H = 0.93-0.98Å, O—H = 0.82Å) and refined as riding with U_iso_(H) = 1.2U_eq_(C) or 1.5U_eq_(methyl C, O).

### Crystal data

**Table:**

C_11_H_18_O_3_	*F*_000_ = 432
*M**_r_* = 198.25	*D*_x_ = 1.103 Mg m^−^^3^
Monoclinic, *P*2_1_	Mo *K*α radiation λ = 0.71073 Å
Hall symbol: P 2yb	Cell parameters from 9263 reflections
*a* = 6.2934 (13) Å	θ = 3.0–25.4º
*b* = 16.411 (3) Å	µ = 0.08 mm^−^^1^
*c* = 11.607 (2) Å	*T* = 291 (1) K
β = 95.46 (3)º	Block, colourless
*V* = 1193.4 (4) Å^3^	0.30 × 0.28 × 0.20 mm
*Z* = 4	

### Data collection

**Table:**

Nonius KappaCCD diffractometer	2268 independent reflections
Radiation source: fine-focus sealed tube	1228 reflections with *I* > 2σ(*I*)
Monochromator: graphite	*R*_int_ = 0.026
Detector resolution: 19 vertical, 18 horizontal pixels mm^-1^	θ_max_ = 25.4º
*T* = 291(1) K	θ_min_ = 3.0º
259 frames via ω–rotation (Δω=1%) and two times 120 s per frame (three sets at different κ–angles) scans	*h* = −7→7
Absorption correction: none	*k* = −19→19
9263 measured reflections	*l* = −13→13

### Refinement

**Table:**

Refinement on *F*^2^	Secondary atom site location: difference Fourier map
Least-squares matrix: full	Hydrogen site location: inferred from neighbouring sites
*R*[*F*^2^ > 2σ(*F*^2^)] = 0.036	H-atom parameters constrained
*wR*(*F*^2^) = 0.080	*w* = 1/[σ^2^(*F*_o_^2^) + (0.0276*P*)^2^] where *P* = (*F*_o_^2^ + 2*F*_c_^2^)/3
*S* = 1.06	(Δ/σ)_max_ = 0.001
2268 reflections	Δρ_max_ = 0.14 e Å^−^^3^
262 parameters	Δρ_min_ = −0.10 e Å^−^^3^
1 restraint	Extinction correction: SHELXL97 (Sheldrick, 2008), Fc^*^=kFc[1+0.001xFc^2^λ^3^/sin(2θ)]^-1/4^
Primary atom site location: structure-invariant direct methods	Extinction coefficient: 0.042 (4)

### Special details

**Table:**

Geometry. All e.s.d.'s (except the e.s.d. in the dihedral angle between two l.s. planes) are estimated using the full covariance matrix. The cell e.s.d.'s are taken into account individually in the estimation of e.s.d.'s in distances, angles and torsion angles; correlations between e.s.d.'s in cell parameters are only used when they are defined by crystal symmetry. An approximate (isotropic) treatment of cell e.s.d.'s is used for estimating e.s.d.'s involving l.s. planes.
Refinement. Refinement of *F*^2^ against ALL reflections. The weighted *R*-factor *wR* and goodness of fit *S* are based on *F*^2^, conventional *R*-factors *R* are based on *F*, with *F* set to zero for negative *F*^2^. The threshold expression of *F*^2^ > σ(*F*^2^) is used only for calculating *R*-factors(gt) *etc*. and is not relevant to the choice of reflections for refinement. *R*-factors based on *F*^2^ are statistically about twice as large as those based on *F*, and *R*- factors based on ALL data will be even larger.

### Fractional atomic coordinates and isotropic or equivalent isotropic displacement parameters (Å^2^)

**Table:**

	*x*	*y*	*z*	*U*_iso_*/*U*_eq_	
O1	−0.2547 (2)	0.96841 (13)	0.63254 (16)	0.0723 (6)	
O2	0.1616 (3)	0.91394 (14)	0.55403 (19)	0.0818 (7)	
H2	0.2747	0.9160	0.5245	0.123*	
O3	−0.4523 (3)	0.95694 (15)	0.46477 (16)	0.0804 (6)	
C1	0.0831 (4)	0.99366 (19)	0.5670 (2)	0.0699 (9)	
H1	0.1979	1.0342	0.5700	0.084*	
C2	−0.0400 (4)	0.9980 (2)	0.6745 (2)	0.0717 (9)	
H2A	−0.0512	1.0552	0.6971	0.086*	
C3	−0.2854 (5)	0.9770 (2)	0.5177 (3)	0.0677 (8)	
C4	−0.0916 (4)	1.0127 (2)	0.4718 (2)	0.0713 (9)	
H4	−0.1101	1.0720	0.4700	0.086*	
C5	−0.0562 (5)	0.9858 (2)	0.3503 (2)	0.0963 (11)	
H5A	−0.0272	0.9284	0.3501	0.144*	
H5B	0.0629	1.0149	0.3246	0.144*	
H5C	−0.1818	0.9971	0.2992	0.144*	
C6	0.0466 (4)	0.9499 (2)	0.7790 (2)	0.0766 (9)	
H6	0.0636	0.8934	0.7541	0.092*	
C7	0.2689 (5)	0.9825 (2)	0.8203 (3)	0.0999 (12)	
H7A	0.3668	0.9687	0.7647	0.150*	
H7B	0.3165	0.9585	0.8936	0.150*	
H7C	0.2628	1.0406	0.8283	0.150*	
C8	−0.1058 (5)	0.9493 (3)	0.8754 (3)	0.0969 (12)	
H8	−0.2441	0.9299	0.8397	0.116*	
C9	−0.1446 (7)	1.0344 (3)	0.9243 (3)	0.1352 (16)	
H9A	−0.2459	1.0306	0.9809	0.203*	
H9B	−0.1995	1.0698	0.8626	0.203*	
H9C	−0.0125	1.0560	0.9598	0.203*	
C10	−0.0295 (7)	0.8869 (4)	0.9651 (4)	0.146 (2)	
H10	0.0090	0.8378	0.9327	0.175*	
C11	−0.0089 (10)	0.8863 (6)	1.0604 (6)	0.278 (5)	
H11A	−0.0428	0.9323	1.1017	0.333*	
H11B	0.0422	0.8398	1.0995	0.333*	
O1'	−0.6701 (3)	0.71545 (13)	0.46394 (18)	0.0769 (6)	
O2'	−0.2446 (3)	0.67928 (15)	0.3828 (2)	0.0935 (7)	
H2'	−0.1359	0.6868	0.3505	0.140*	
O3'	−0.8637 (4)	0.71727 (16)	0.29367 (18)	0.0963 (8)	
C1'	−0.3349 (5)	0.7549 (2)	0.4080 (3)	0.0777 (9)	
H1'	−0.2255	0.7974	0.4203	0.093*	
C2'	−0.4615 (4)	0.7458 (2)	0.5138 (3)	0.0727 (9)	
H2'1	−0.4816	0.8000	0.5465	0.087*	
C3'	−0.7005 (5)	0.7353 (2)	0.3510 (3)	0.0794 (10)	
C4'	−0.5091 (5)	0.7794 (2)	0.3145 (3)	0.0839 (10)	
H4'	−0.5347	0.8379	0.3232	0.101*	
C5'	−0.4680 (6)	0.7646 (3)	0.1898 (3)	0.1211 (15)	
H5'1	−0.3427	0.7940	0.1730	0.182*	
H5'2	−0.5883	0.7831	0.1396	0.182*	
H5'3	−0.4468	0.7074	0.1779	0.182*	
C6'	−0.3719 (4)	0.6903 (2)	0.6094 (3)	0.0726 (9)	
H6'	−0.3515	0.6367	0.5749	0.087*	
C7'	−0.1534 (5)	0.7211 (2)	0.6567 (3)	0.0986 (12)	
H7'1	−0.0566	0.7169	0.5978	0.148*	
H7'2	−0.1013	0.6888	0.7224	0.148*	
H7'3	−0.1642	0.7770	0.6797	0.148*	
C8'	−0.5233 (5)	0.6788 (2)	0.7046 (3)	0.0773 (9)	
H8'	−0.6609	0.6623	0.6647	0.093*	
C9'	−0.5664 (6)	0.7563 (3)	0.7697 (3)	0.1204 (14)	
H9'1	−0.4354	0.7760	0.8091	0.181*	
H9'2	−0.6664	0.7450	0.8252	0.181*	
H9'3	−0.6248	0.7968	0.7161	0.181*	
C10'	−0.4516 (6)	0.6101 (3)	0.7795 (4)	0.1110 (13)	
H10'	−0.4328	0.5614	0.7408	0.133*	
C11'	−0.4126 (8)	0.6067 (4)	0.8851 (4)	0.183 (3)	
H11C	−0.4276	0.6530	0.9300	0.220*	
H11D	−0.3679	0.5579	0.9203	0.220*	

### Atomic displacement parameters (Å^2^)

**Table:**

	*U*^11^	*U*^22^	*U*^33^	*U*^12^	*U*^13^	*U*^23^
O1	0.0470 (10)	0.0854 (17)	0.0864 (13)	−0.0022 (11)	0.0168 (9)	0.0018 (13)
O2	0.0649 (13)	0.0683 (17)	0.1164 (16)	0.0050 (11)	0.0310 (12)	0.0017 (13)
O3	0.0569 (12)	0.0926 (18)	0.0930 (13)	−0.0018 (13)	0.0134 (11)	0.0007 (13)
C1	0.0543 (17)	0.052 (2)	0.106 (2)	−0.0006 (16)	0.0204 (16)	−0.0009 (18)
C2	0.0513 (16)	0.072 (3)	0.094 (2)	−0.0065 (16)	0.0167 (15)	−0.0091 (19)
C3	0.0549 (18)	0.063 (2)	0.088 (2)	0.0086 (17)	0.0190 (16)	0.0012 (19)
C4	0.0609 (18)	0.065 (2)	0.090 (2)	0.0004 (16)	0.0210 (16)	0.0089 (17)
C5	0.083 (2)	0.112 (3)	0.099 (2)	−0.003 (2)	0.0322 (17)	0.004 (2)
C6	0.0509 (16)	0.091 (3)	0.0888 (19)	−0.0027 (17)	0.0115 (15)	−0.007 (2)
C7	0.0603 (18)	0.125 (4)	0.115 (2)	−0.008 (2)	0.0097 (16)	−0.012 (2)
C8	0.0648 (19)	0.140 (4)	0.087 (2)	−0.003 (2)	0.0156 (18)	−0.007 (3)
C9	0.111 (3)	0.173 (5)	0.128 (3)	0.024 (3)	0.047 (3)	−0.021 (3)
C10	0.106 (3)	0.249 (7)	0.086 (3)	0.007 (4)	0.027 (3)	0.030 (4)
C11	0.147 (5)	0.476 (16)	0.222 (7)	0.051 (7)	0.075 (6)	0.165 (9)
O1'	0.0496 (11)	0.0897 (18)	0.0938 (14)	−0.0022 (11)	0.0186 (10)	0.0066 (12)
O2'	0.0718 (14)	0.0719 (18)	0.1435 (19)	0.0044 (13)	0.0446 (13)	0.0045 (15)
O3'	0.0646 (14)	0.125 (2)	0.1002 (15)	−0.0110 (15)	0.0131 (12)	0.0065 (14)
C1'	0.0634 (19)	0.056 (2)	0.117 (2)	−0.0012 (17)	0.0241 (19)	0.003 (2)
C2'	0.0534 (18)	0.060 (2)	0.106 (2)	−0.0041 (16)	0.0122 (16)	−0.003 (2)
C3'	0.060 (2)	0.086 (3)	0.095 (2)	0.0041 (19)	0.0236 (18)	0.006 (2)
C4'	0.073 (2)	0.069 (3)	0.112 (2)	−0.0002 (18)	0.0255 (19)	0.011 (2)
C5'	0.103 (3)	0.153 (4)	0.114 (3)	−0.008 (3)	0.045 (2)	0.016 (3)
C6'	0.0542 (17)	0.064 (2)	0.101 (2)	0.0017 (16)	0.0160 (16)	−0.005 (2)
C7'	0.0560 (18)	0.097 (3)	0.142 (3)	−0.0047 (19)	0.0043 (18)	−0.004 (2)
C8'	0.0616 (18)	0.079 (3)	0.092 (2)	0.0030 (18)	0.0084 (16)	0.002 (2)
C9'	0.107 (3)	0.128 (4)	0.128 (3)	0.021 (3)	0.019 (2)	−0.026 (3)
C10'	0.078 (2)	0.149 (4)	0.108 (3)	0.001 (3)	0.020 (2)	0.015 (3)
C11'	0.134 (4)	0.252 (7)	0.161 (4)	0.028 (4)	−0.001 (4)	0.069 (5)

### Geometric parameters (Å, °)

**Table:**

O1—C3	1.336 (3)	O1'—C3'	1.347 (3)
O1—C2	1.474 (3)	O1'—C2'	1.470 (3)
O2—C1	1.411 (3)	O2'—C1'	1.408 (4)
O2—H2	0.8200	O2'—H2'	0.8200
O3—C3	1.211 (3)	O3'—C3'	1.206 (3)
C1—C4	1.515 (4)	C1'—C4'	1.521 (4)
C1—C2	1.532 (4)	C1'—C2'	1.533 (4)
C1—H1	0.9800	C1'—H1'	0.9800
C2—C6	1.504 (4)	C2'—C6'	1.503 (4)
C2—H2A	0.9800	C2'—H2'1	0.9800
C3—C4	1.496 (4)	C3'—C4'	1.500 (5)
C4—C5	1.515 (4)	C4'—C5'	1.514 (4)
C4—H4	0.9800	C4'—H4'	0.9800
C5—H5A	0.9600	C5'—H5'1	0.9600
C5—H5B	0.9600	C5'—H5'2	0.9600
C5—H5C	0.9600	C5'—H5'3	0.9600
C6—C7	1.531 (4)	C6'—C7'	1.517 (4)
C6—C8	1.542 (4)	C6'—C8'	1.538 (4)
C6—H6	0.9800	C6'—H6'	0.9800
C7—H7A	0.9600	C7'—H7'1	0.9600
C7—H7B	0.9600	C7'—H7'2	0.9600
C7—H7C	0.9600	C7'—H7'3	0.9600
C8—C10	1.506 (7)	C8'—C10'	1.469 (6)
C8—C9	1.536 (6)	C8'—C9'	1.516 (5)
C8—H8	0.9800	C8'—H8'	0.9800
C9—H9A	0.9600	C9'—H9'1	0.9600
C9—H9B	0.9600	C9'—H9'2	0.9600
C9—H9C	0.9600	C9'—H9'3	0.9600
C10—C11	1.102 (6)	C10'—C11'	1.228 (5)
C10—H10	0.9300	C10'—H10'	0.9300
C11—H11A	0.9300	C11'—H11C	0.9300
C11—H11B	0.9300	C11'—H11D	0.9300
			
C3—O1—C2	109.6 (2)	C3'—O1'—C2'	109.9 (2)
C1—O2—H2	109.5	C1'—O2'—H2'	109.5
O2—C1—C4	110.5 (3)	O2'—C1'—C4'	111.2 (3)
O2—C1—C2	109.8 (2)	O2'—C1'—C2'	109.3 (3)
C4—C1—C2	101.3 (2)	C4'—C1'—C2'	101.7 (2)
O2—C1—H1	111.6	O2'—C1'—H1'	111.4
C4—C1—H1	111.6	C4'—C1'—H1'	111.4
C2—C1—H1	111.6	C2'—C1'—H1'	111.4
O1—C2—C6	110.1 (2)	O1'—C2'—C6'	110.1 (2)
O1—C2—C1	103.6 (2)	O1'—C2'—C1'	103.3 (2)
C6—C2—C1	117.4 (2)	C6'—C2'—C1'	117.6 (2)
O1—C2—H2A	108.5	O1'—C2'—H2'1	108.5
C6—C2—H2A	108.5	C6'—C2'—H2'1	108.5
C1—C2—H2A	108.5	C1'—C2'—H2'1	108.5
O3—C3—O1	120.9 (2)	O3'—C3'—O1'	120.8 (3)
O3—C3—C4	128.5 (3)	O3'—C3'—C4'	128.8 (3)
O1—C3—C4	110.6 (3)	O1'—C3'—C4'	110.4 (3)
C3—C4—C5	114.5 (3)	C3'—C4'—C5'	114.1 (3)
C3—C4—C1	102.7 (2)	C3'—C4'—C1'	102.4 (3)
C5—C4—C1	117.3 (2)	C5'—C4'—C1'	117.5 (3)
C3—C4—H4	107.3	C3'—C4'—H4'	107.4
C5—C4—H4	107.3	C5'—C4'—H4'	107.4
C1—C4—H4	107.3	C1'—C4'—H4'	107.4
C4—C5—H5A	109.5	C4'—C5'—H5'1	109.5
C4—C5—H5B	109.5	C4'—C5'—H5'2	109.5
H5A—C5—H5B	109.5	H5'1—C5'—H5'2	109.5
C4—C5—H5C	109.5	C4'—C5'—H5'3	109.5
H5A—C5—H5C	109.5	H5'1—C5'—H5'3	109.5
H5B—C5—H5C	109.5	H5'2—C5'—H5'3	109.5
C2—C6—C7	108.6 (3)	C2'—C6'—C7'	109.2 (3)
C2—C6—C8	112.7 (3)	C2'—C6'—C8'	113.0 (2)
C7—C6—C8	112.9 (2)	C7'—C6'—C8'	112.5 (3)
C2—C6—H6	107.5	C2'—C6'—H6'	107.3
C7—C6—H6	107.5	C7'—C6'—H6'	107.3
C8—C6—H6	107.5	C8'—C6'—H6'	107.3
C6—C7—H7A	109.5	C6'—C7'—H7'1	109.5
C6—C7—H7B	109.5	C6'—C7'—H7'2	109.5
H7A—C7—H7B	109.5	H7'1—C7'—H7'2	109.5
C6—C7—H7C	109.5	C6'—C7'—H7'3	109.5
H7A—C7—H7C	109.5	H7'1—C7'—H7'3	109.5
H7B—C7—H7C	109.5	H7'2—C7'—H7'3	109.5
C10—C8—C9	114.5 (3)	C10'—C8'—C9'	114.0 (3)
C10—C8—C6	109.1 (3)	C10'—C8'—C6'	110.1 (3)
C9—C8—C6	113.1 (3)	C9'—C8'—C6'	114.1 (3)
C10—C8—H8	106.5	C10'—C8'—H8'	106.0
C9—C8—H8	106.5	C9'—C8'—H8'	106.0
C6—C8—H8	106.5	C6'—C8'—H8'	106.0
C8—C9—H9A	109.5	C8'—C9'—H9'1	109.5
C8—C9—H9B	109.5	C8'—C9'—H9'2	109.5
H9A—C9—H9B	109.5	H9'1—C9'—H9'2	109.5
C8—C9—H9C	109.5	C8'—C9'—H9'3	109.5
H9A—C9—H9C	109.5	H9'1—C9'—H9'3	109.5
H9B—C9—H9C	109.5	H9'2—C9'—H9'3	109.5
C11—C10—C8	134.4 (8)	C11'—C10'—C8'	130.3 (5)
C11—C10—H10	112.8	C11'—C10'—H10'	114.9
C8—C10—H10	112.8	C8'—C10'—H10'	114.9
C10—C11—H11A	120.0	C10'—C11'—H11C	120.0
C10—C11—H11B	120.0	C10'—C11'—H11D	120.0
H11A—C11—H11B	120.0	H11C—C11'—H11D	120.0
			
C3—O1—C2—C6	−148.1 (3)	C3'—O1'—C2'—C6'	−149.0 (3)
C3—O1—C2—C1	−21.8 (3)	C3'—O1'—C2'—C1'	−22.6 (3)
O2—C1—C2—O1	−83.7 (3)	O2'—C1'—C2'—O1'	−84.1 (3)
C4—C1—C2—O1	33.2 (3)	C4'—C1'—C2'—O1'	33.5 (3)
O2—C1—C2—C6	37.9 (3)	O2'—C1'—C2'—C6'	37.4 (4)
C4—C1—C2—C6	154.7 (3)	C4'—C1'—C2'—C6'	155.1 (3)
C2—O1—C3—O3	−179.6 (3)	C2'—O1'—C3'—O3'	−178.9 (3)
C2—O1—C3—C4	0.5 (3)	C2'—O1'—C3'—C4'	1.6 (3)
O3—C3—C4—C5	−30.6 (5)	O3'—C3'—C4'—C5'	−31.2 (6)
O1—C3—C4—C5	149.3 (3)	O1'—C3'—C4'—C5'	148.3 (3)
O3—C3—C4—C1	−158.8 (3)	O3'—C3'—C4'—C1'	−159.3 (4)
O1—C3—C4—C1	21.1 (3)	O1'—C3'—C4'—C1'	20.2 (4)
O2—C1—C4—C3	84.0 (3)	O2'—C1'—C4'—C3'	84.1 (3)
C2—C1—C4—C3	−32.4 (3)	C2'—C1'—C4'—C3'	−32.2 (3)
O2—C1—C4—C5	−42.5 (4)	O2'—C1'—C4'—C5'	−41.7 (4)
C2—C1—C4—C5	−158.9 (3)	C2'—C1'—C4'—C5'	−158.0 (3)
O1—C2—C6—C7	−179.8 (2)	O1'—C2'—C6'—C7'	178.0 (2)
C1—C2—C6—C7	62.1 (4)	C1'—C2'—C6'—C7'	60.1 (4)
O1—C2—C6—C8	−54.0 (4)	O1'—C2'—C6'—C8'	−56.0 (4)
C1—C2—C6—C8	−172.1 (3)	C1'—C2'—C6'—C8'	−173.9 (3)
C2—C6—C8—C10	168.6 (3)	C2'—C6'—C8'—C10'	166.5 (3)
C7—C6—C8—C10	−67.9 (5)	C7'—C6'—C8'—C10'	−69.3 (4)
C2—C6—C8—C9	−62.7 (4)	C2'—C6'—C8'—C9'	−63.8 (4)
C7—C6—C8—C9	60.8 (4)	C7'—C6'—C8'—C9'	60.4 (4)
C9—C8—C10—C11	7.2 (9)	C9'—C8'—C10'—C11'	−3.6 (7)
C6—C8—C10—C11	135.1 (8)	C6'—C8'—C10'—C11'	126.1 (5)

### Hydrogen-bond geometry (Å, °)

**Table:**

*D*—H···*A*	*D*—H	H···*A*	*D*···*A*	*D*—H···*A*
O2—H2···O3^i^	0.82	2.03	2.821 (3)	163
O2'—H2'···O3'^i^	0.82	1.96	2.771 (3)	171

Symmetry codes: (i) *x*+1, *y*, *z*.
